# Assistive Artificial Intelligence in Epilepsy and Its Impact on Epilepsy Care in Low- and Middle-Income Countries

**DOI:** 10.3390/brainsci15050481

**Published:** 2025-05-01

**Authors:** Nabin Koirala, Shishir Raj Adhikari, Mukesh Adhikari, Taruna Yadav, Abdul Rauf Anwar, Dumitru Ciolac, Bibhusan Shrestha, Ishan Adhikari, Bishesh Khanal, Muthuraman Muthuraman

**Affiliations:** 1 School of Medicine, Yale University, New Haven, CT 06511, USA; taruna.yadav@yale.edu; 2 Brain Imaging Research Core, University of Connecticut, Storrs, CT 06269, USA; 3 Nathan Kline Institute for Psychiatric Research, Orangeburg, NY 10962, USA; 4 Nepal Applied Mathematics and Informatics Institute for Research, Kathmandu 44700, Nepal; adhikarirshishir@gmail.com (S.R.A.); bishesh.khanal@naamii.org.np (B.K.); 5 Gilling’s School of Global Public Health, University of North Carolina, Chapel Hill, NC 27599, USA; madhikari@unc.edu; 6 Institut du Cerveau—Paris Brain Institute, 75013 Paris, France; raufanwar@gmail.com; 7 Department of Neurology, State University of Medicine and Pharmacy “Nicolae Testemitanu”, MD-2004 Chisinau, Moldova; dimaciolac@gmail.com; 8 Department of Surgery, Kathmandu University Hospital, Dhulikhel 45200, Nepal; sbibhusan@hotmail.com; 9 Department of Neurology, University of Texas, San Antonio, TX 78249, USA; ishan.adhikari@gmail.com; 10 Neural Engineering with Signal Analytics and Artificial Intelligence, Department of Neurology, University of Wurzburg, 97070 Wurzburg, Germany; 11 Informatics for Medical Technology, Institute of Computer Science, University of Augsburg, 86159 Augsburg, Germany

**Keywords:** epilepsy, epilepsy eiagnosis, epilepsy care, artificial intelligence, low and middle income countries

## Abstract

Epilepsy, one of the most common neurological diseases in the world, affects around 50 million people, with a notably disproportionate prevalence in individuals residing in low- and middle-income countries (LMICs). Alarmingly, over 80% of annual epilepsy-related fatalities occur within LMICs. The burden of the disease assessed using Disability Adjusted Life Years (DALYs) shows that epilepsy accounts for about 13 million DALYs per year, with LMICs bearing most of this burden due to the disproportionately high diagnostic and treatment gaps. Furthermore, LMICs also endure a significant financial burden, with the cost of epilepsy reaching up to 0.5% of the Gross National Product (GNP) in some cases. Difficulties in the appropriate diagnosis and treatment are complicated by the lack of trained medical specialists. Therefore, in these conditions, adopting artificial intelligence (AI)-based solutions may improve epilepsy care in LMICs. In this theoretical and critical review, we focus on epilepsy and its management in LMICs, as well as on the employment of AI technologies to aid epilepsy care in LMICs. We begin with a general introduction of epilepsy and present basic diagnostic and treatment approaches. We then explore the socioeconomic impact, treatment gaps, and efforts made to mitigate these issues. Taking this step further, we examine recent AI-related developments and their potential as assistive tools in clinical application in LMICs, along with proposals for future directions. We conclude by suggesting the need for scalable, low-cost AI solutions that align with the local infrastructure, policy and community engagement to improve epilepsy care in LMICs.

## 1. Introduction

An epileptic seizure is defined as the transient occurrence of signs and/or symptoms due to sudden and abnormally excessive neuronal activity in the brain [1]. Epilepsy, often referred to as a seizure disorder, is a neurological condition characterized by a long-lasting predisposition to recurring epileptic seizures [2]. Approximately 50 million people worldwide (about 1.7 times the population of Nepal) are affected by epilepsy, resulting in approximately 125,000 deaths per year, with over 80% of these occurring in low- and middle-income countries (LMICs) [3,4]. Seizures should be differentiated from nonepileptic events, which may arise from psychological, neurological, or physical causes such as emotional stress, anxiety, migraine, stroke, or traumatic brain injury. [5,6]. An epileptic seizure may cause convulsions, muscle spasms, loss of consciousness, and changes in behavior, sensation, or cognition, depending on its type and location in the brain [7].

**Figure 1:**
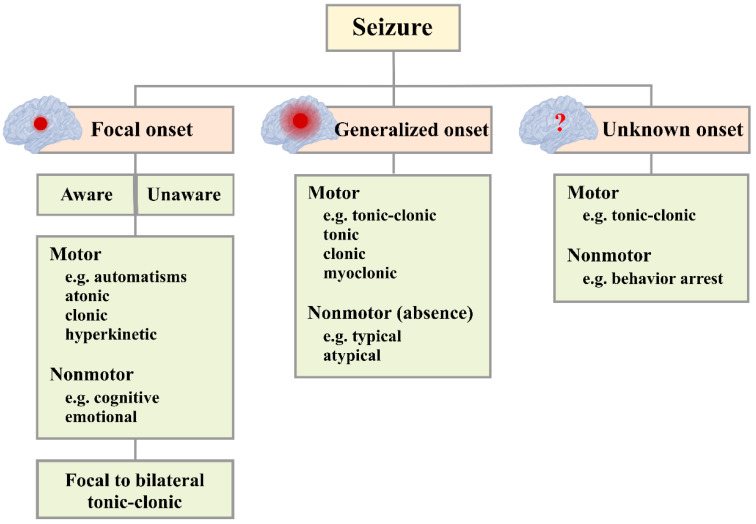
Seizure classification (modified from [8]).

The International League Against Epilepsy has established a classification system for seizures based on their onset [8], as depicted in Figure 1. *Focal onset seizures*: These seizures originate in a localized region of the cerebral cortex within one hemisphere of the brain. Focal seizures are further categorized based on the individual’s level of consciousness during the event [8]. In focal aware seizures, the individual remains conscious and alert throughout the episode. Conversely, focal impaired awareness seizures are characterized by a disturbance in consciousness, during which the individual may experience confusion or an altered state of awareness [9]. *Generalized onset seizures*: These seizures are also characterized by their localized onset but with the subsequent rapid and simultaneous involvement of multiple brain regions [8]. Generalized seizures are further classified into various subtypes, including tonic (muscle stiffness), clonic (repeated jerking movements), tonic–clonic (grand mal seizures, involving a combination of muscle stiffness and jerking), atonic (loss of muscle control), myoclonic (sudden muscle jerks), and absence seizures (petit mal, brief lapses in awareness) [9]. *Unknown onset seizures:* This category pertains to seizures whose origin cannot be determined or was not observed [8]. These seizures are classified as unknown onset due to the absence of direct witnesses or insufficient evidence to ascertain their precise point of origin within the brain [9].

***Diagnosis.*** Epilepsy is diagnosed through a combination of medical history assessments, neurological and neuropsychological examinations, and a series of diagnostic tests [10]. Within the spectrum of diagnostic methods, electroencephalography (EEG) is particularly notable in LMICs due to its economic viability and portability. EEG is a diagnostic modality that quantitatively records the brain’s electrical activity [11]. This procedure involves the placement of electrodes on the scalp to capture the summated electrical activity of numerous neurons firing synchronously, thereby providing a population-level signal measurement. EEG plays a central role in resolving key inquiries in the diagnostic process for individuals suspected of manifesting epileptic seizures [11]. In particular, it can help us answer the following two questions: (1) *Does the patient exhibit clinical manifestations consistent with epilepsy*? Interictal epileptiform discharges (IEDs) are episodes of spike or sharp wave activity observable on the EEG during the interictal interval—the period between seizures [11]. IEDs can appear in various patterns, including spikes, sharp waves, spike–wave complexes, polyspikes, and hypsarrhythmia [11]. Their identification via EEG is pivotal in distinguishing epileptic seizures from non-epileptic events. While the occurrence of IEDs in individuals without epilepsy is rare, their prevalence can reach up to 98% in certain demographic groups diagnosed with epilepsy [12]. Nevertheless, the presence of IEDs, though strongly suggestive, is not sufficient for a definitive diagnosis, and a comprehensive evaluation incorporating clinical history and additional diagnostic findings is required [12]. (2) *Where is the epileptogenic zone*? EEG during the initial evaluation is also essential for localizing the epileptogenic zone—the region of the brain responsible for seizure generation [13]. The analysis of signal patterns and recordings provides valuable clues regarding the seizure onset location [12]. Moreover, specific patterns are often associated with epilepsy syndromes, offering valuable insights into the type of epilepsy. For instance, the presence of 3 Hz spike–wave complexes can indicate absence epilepsy [14]. Notably, ictal video-EEG recordings, which combine video recordings of seizures with simultaneous EEG data acquisition, play a crucial role in localization [14]. By analyzing the initial seizure semiology alongside EEG onset patterns, this technique can effectively pinpoint the epileptogenic region in approximately 72% of cases [13].

While EEG is highly effective in diagnosing epilepsy, it is less useful for monitoring the effectiveness of anti-seizure medications (ASMs) [12]. Moreover, the manual examination of EEG signals by specialists is time-consuming and prone to human error, potentially affecting diagnostic accuracy and reliability. These limitations underscore the need for a robust, computer-based diagnostic system. In this context, a highly trained clinical neurophysiologist or epileptologist would play a crucial role in developing, validating, and operating such systems to ensure clinical applicability and precision.

Epilepsy care in LMICs faces numerous challenges, among which the limited availability of diagnostic tools is a major issue. Therefore, the purpose of this review is to provide an overview of epilepsy care in LMICs and to propose reliable solutions aimed at improving the quality of care for epilepsy patients. We posit that artificial intelligence (AI)-based tools may serve as substantial support in addressing these existing gaps in epilepsy care and deliver more effective care to these patients.

## 2. Current State of Epilepsy in LMICs

At any given time, the global prevalence of active epilepsy—defined as individuals experiencing ongoing seizures or requiring treatment—is approximately 4 to 10 per 1000 people. Additionally, approximately 5 million people are diagnosed with epilepsy worldwide each year [15,16,17]. Importantly, the distribution of epilepsy is highly disproportionate across LMICs [4,18]. Table 1 highlights the stark contrasts in epilepsy-related metrics between high-income countries (HICs) and LMICs, underscoring the significantly greater burden of epilepsy in LMICs.

***Socio-economic impact.*** The economic impact of epilepsy is substantial. A recent estimate indicates that the total global cost of epilepsy in 2019 was $119.27 billion, with the annual cost per patient ranging from $204 in LMICs to $11,432 in HICs [19]. This discrepancy in average cost is largely attributed to the higher treatment gap in LMICs, which ranges from 25% to 100%, compared to only about 10% in HICs [20,21]. Although the per-person cost is higher in HICs, approximately 90% of the total financial burden of epilepsy is borne by LMICs [22,23]. Moreover, the cost of epilepsy in LMICs is disproportionately high relative to the Gross National Product (GNP). For instance, one estimate suggests that epilepsy accounts for 0.5% of India’s annual GNP [22]. From the patient’s perspective, the costs of diagnosis and treatment can be financially catastrophic. For instance, 50% of epileptic children attending a tertiary care center in Nigeria incurred out-of-pocket expenditures equivalent to 20% of their annual household income [24].

In addition to the economic burden, the social costs associated with stigma for epilepsy patients are significant and widespread [25], which at times can be even greater than that associated with acquired immunodeficiency syndrome (AIDS) [26]. Due to persistent myths, misconceptions, and cultural misunderstandings, people with epilepsy often face discrimination, including social rejection, public disapproval, shaming, and employment-related stigma or job loss [27,28,29]. More critically, internalized or perceived stigma—experienced as shame or embarrassment—can be particularly detrimental and may hinder treatment-seeking behavior [30,31,32]. Such challenges are more common in LMICs compared to HICs. For instance, multiple civil and human rights violations against people with epilepsy have been reported in developing countries, many of which lacks legislation to protect against such discrimination [33,34]. In addition to its seizure-related symptoms, epilepsy is also reported to be the contributing factor to psychological distress in almost 70% of its patients [35]. This leads to a “double stigma” scenario, wherein individuals with epilepsy also suffer from comorbid psychological conditions such as depression or anxiety—further compounding the personal and societal burden [36].

***Access to treatment and diagnosis.*** Most people with epilepsy reside in LMICs; however, on average, 50 to 70% of them are deprived of treatment—a phenomenon known as the epilepsy treatment gap (ETG) [4]. The ETG refers to the percentage of individuals with active epilepsy who either do not have access to treatment or receive inadequate treatment at a given point in time within a specific population. Alarmingly, in some LMICs such as Tibet, Togo, and Uganda, the ETG approaches 100% [37]. Moreover, in many LMICs, the ETG varies drastically between rural and urban areas. For example, in Pakistan, the prevalence of epilepsy is 0.98%, with an ETG of 98% in rural compared to 75% in the urban population [38]. This higher rate of ETG in LMICs is multifactorial. First, anti-epileptic drugs (AEDs) are often not adequately available. Data from 46 LMICs showed that more than half of public sector pharmacies did not have any of the five AEDs surveyed, except diazepam [39]. Even when AEDs are available, people with epilepsy in LMICs face challenges associated with accessibility to health service facilities. For instance, the recommended advice to have a brain scan to rule out any underlying brain lesion that might be the cause of the seizures is not possible in many of these countries; as a result, the diagnosis and treatment course often relies on the patient’s history and clinician’s experience [40,41,42]. Affordability is another significant barrier. In the same survey of 46 LMICs, the prices for generic carbamazepine and phenytoin in the public sector were 5 to 17.5 times higher than international reference prices, which translates to a financial burden equivalent to 2.6 to 16.2 days of wages for a monthly supply [39]. The low level of awareness about treatment, including misconceptions and fear of stigmatization, has further exacerbate the treatment gap [43]. Additionally, the lack of neurologists and epileptologists and the limited availability of epilepsy surgery, needed by approximately 20–30% of patients who do not respond to AEDs, contribute to the high ETG in LMICs [44,45].

In addition to the treatment gap, the underdiagnosis and misdiagnosis of epilepsy are other major challenges in LMICs. This is mainly due to the limited availability of diagnostic tools and the scarcity of neurologists and epileptologists [46]. For example, the *Atlas of Epilepsy Care in the World*, published in 2005, showed that only 21.7% of low-income countries and 20.6% of LMICs in Africa had long-term video EEG and MRI, respectively [47]. Similarly, another study showed that a major cause of epilepsy in Nepal was neurocysticercosis, a preventable infection from pork tapeworm larval cysts. However, many Nepalese attribute epilepsy to a supernatural origin and seek traditional remedies instead of modern medical care [48]. Therefore, the combination of lack of awareness and proper diagnostics tools are some of the biggest hurdles in overcoming the misdiagnosis and underdiagnosis of epilepsy in LMICs.

***Efforts in compensating the access to treatment and diagnosis.*** Over the years, several initiatives have been made to overcome some of these issues. To reduce the reliance on specialists analyzing EEG data, a group of researchers in Colombia developed an intelligent system to automatically detect, annotate, and visualize the abnormal segments of EEG tests using machine learning algorithms [49]. Additionally, the algorithm could evaluate intelligent components and compute various metrics to assist clinicians, which could then be added to patients’ records. Similarly, a method for the automatic detection of epileptic seizures in long-term scalp-EEG recordings, called *EpiScan,* was developed and tested in a multi-center study; it was used as an alarm system to notify medical staff in epilepsy monitoring units in the case of a seizure [50]. Another study highlighted the lack of trained health professionals in the majority of LMIC areas and proposed training non-physician health workers for basic epilepsy care in public health settings [51]. The study also reviewed the telemedicine approaches for epilepsy diagnosis and management and suggested improvements for making it more accessible and efficient. The same group of researchers developed a mobile phone-based application that uses the Bayesian approach to quantify the likelihood of the patient having an epileptic seizure based on responses to 50 routinely asked questions [52]. This application was validated using non-physician health workers implementing the telemedicine approach in Nepal and Bolivia [53,54,55]. It was later enhanced to classify whether the seizures were epileptic or not, and to further categorize them as focal or generalized [56]. In another multicenter prospective validation study, researchers developed and tested a web-based algorithm capable of accurately classifying seizure types, which can be used for selecting antiseizure medications in adolescents and adults [57]. The algorithm achieved an 83.2% agreement rate with experts’ classification. Moreover, the feasibility of the algorithm was tested by 32 healthcare professionals from 14 countries in their clinical settings, who found it to be both applicable and useful in their practice.

## 3. Artificial Intelligence in Epilepsy

**Figure 2:**
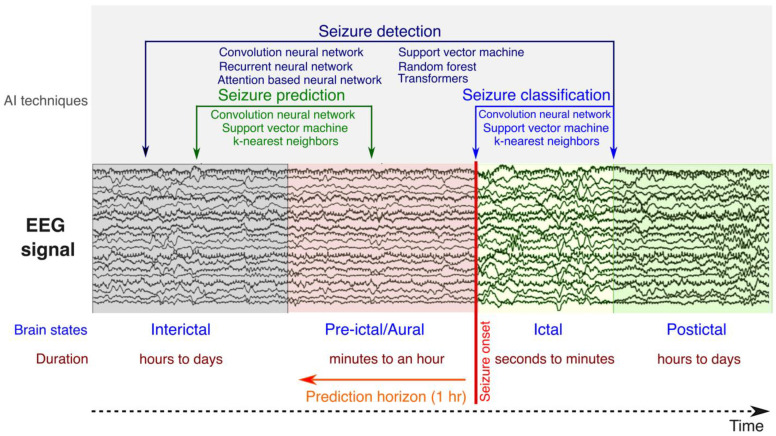
Brain states related to seizure occurrence and the artificial intelligence (AI)-based techniques most frequently applied to predict, detect and classify seizures. Convolutional Neural Networks, Support Vector Machines, and K-Nearest Neighbors are the most popular techniques used for seizure prediction [65]. For seizure detection, one- and two-dimensional convolutional neural networks, Recurrent Neural Networks, Support Vector Machines, and Random Forests are the most widely used models [66], while for seizure classification, convolutional neural network, Support Vector Machine, and K-Nearest Neighbor (KNN) algorithms take precedence.

Recent advancements in artificial intelligence (AI) have significantly impacted the field of epilepsy, enabling breakthroughs in seizure prediction, detection, and classification (Figure 2). By leveraging machine learning algorithms, large datasets, and advanced data analysis techniques, AI systems have been able to provide more accurate and timely insights into seizure episodes. These approaches typically rely on either traditional statistical and machine learning methods, such as Principal Component Analysis, Support Vector Machines, Random Forests, K-Nearest Neighbors, etc. [58], or on more recent deep learning methods [59] like Convolutional Neural Networks (CNNs), Recurrent Neural Networks (RNNs), Long-Short-Term Memory networks (LSTMs) [60], and Transformers [61]. Machine learning, a subfield of AI, encompasses algorithms that learn from data without explicit programming and can make predictions or decisions [62,63]. These methods may be supervised or unsupervised, depending on whether labeled data are available to guide feature extraction. Deep learning, a subset of machine learning, employs artificial neural networks inspired by human brain architecture, and its primary advantage lies in the automated extraction of relevant features from raw data [62,63]. For a more detailed overview of these techniques, readers are referred to the relevant literature [62,63,64].

In the following subsections, we explore each of these critical aspects of diagnosis, prediction, and management.

**Figure 3:**
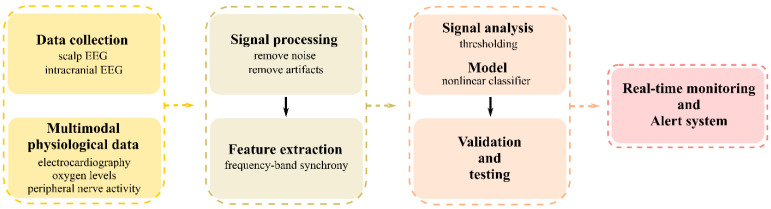
General pipeline of a seizure prediction model.

***Seizure prediction***. The seizure prediction problem refers to the challenge of forecasting epileptic seizures before they occur. This involves identifying patterns or signals in the brain’s electrical activity that precede seizures, known as pre-ictal states, and developing algorithms or systems capable of detecting these signals in real time or near real time [67,68]. A general pipeline for a seizure prediction model is presented in Figure 3. The goal is to provide warnings or interventions that can prevent the seizure, reduce its severity, or alert the patient or caregivers to take precautionary measures. The complexity of predicting a seizure is primarily due to the highly individualized nature of epilepsy and its manifestations in different patients [67]. Typical factors that contribute to the complexity of the seizure prediction include the following:

1. Variability of seizure patterns: Seizures can vary greatly in frequency, duration, and type, not only across individuals but also within the same individual over time [69]. This variability makes it challenging to identify universal predictors or markers that can reliably indicate an impending seizure.
2. Identification of predictive biomarkers: Finding reliable biomarkers (physiological changes or patterns) that consistently precede seizures is crucial for prediction. These biomarkers can include changes in brain electrical activity, as measured by EEG, and other physiological signals [69].
3. Data collection and analysis: Continuous monitoring of brain activity and other physiological signals generates large volumes of data. Analyzing these data requires high computational capacity, sophisticated data processing algorithms, and advanced machine learning techniques [70].
4. Real-time prediction and intervention: For seizure prediction to be clinically relevant, it must operate in real time or near real time, providing timely alerts to patients or triggering interventions to prevent or mitigate the seizure [71]. This necessitates highly accurate prediction algorithms and user-friendly devices for monitoring and intervention.
5. Individualized prediction models: Due to the individual variability in seizure patterns and physiological responses, seizure prediction models often need to be personalized by adding patient-specific information such as medical history and demographics [67]. Developing and tuning these individualized models adds an additional layer of complexity.

Methods for predicting seizures are rapidly evolving, with ongoing research focused on improving prediction accuracy, developing non-invasive monitoring methods, and integrating multimodal data sources [69]. Despite the inherent challenges, recent studies have utilized advancements in computational methods to enable more reliable seizure prediction. For instance, Wei et al. employed multichannel EEG signals and extracted features using a long-term recurrent convolutional network (LRCN) with a long short-term memory (LSTM) block, achieving an accuracy of 93.4% and a sensitivity of 91.88% in predicting pre-ictal segments [72]. Similarly, another study utilized one- and two-dimensional convolutional neural networks (1D and 2D CNNs) and a hybrid model combining approximate entropy with Support Vector Machines (SVMs), where the 2D-CNN achieved an accuracy of 95.2% and specificity of 92.9% for pre-ictal segments [73]. Cousyn et al. applied an SVM-based model to classify pre-ictal and inter-ictal EEG data from patients with drug-resistant epilepsy, yielding an area under the ROC curve of 0.80 (95% CI: 0.69–0.88) [74]. In another comparative study, various machine learning algorithms, including K-Nearest Neighbors (KNNs), Random Forest, Decision Tree, and SVM, were evaluated for seizure detection tasks, with SVM achieving the highest reported accuracy of 98.4% [75].

However, it is important to clarify that these results should not be interpreted as evidence that traditional machine learning models consistently outperform deep learning approaches. In fact, several recent studies have demonstrated superior performance using deep learning methods. For example, Ghaempour et al. reported 98.84% accuracy in seizure detection and 94.29% in prediction using a CNN model on single-lead ECG data [76], while Srinivasan et al. achieved 99.08% accuracy and 99.21% sensitivity using a hybrid deep learning architecture that combines 3D convolutional auto-encoders with a neural network classifier [77]. These findings underscore the growing potential of deep learning methods in enhancing predictive performance, particularly as more sophisticated architectures and larger datasets become available.

***Seizure detection.*** Seizure detection typically refers to the process of identifying epileptic seizures and is a critical component in the management and treatment of epilepsy. This typically involves monitoring brain activity through various diagnostic tests and employing specialized algorithms to recognize patterns indicative of seizures. In the past decade, numerous studies have utilized AI-based methods to discern seizures. Given the heterogeneity of epileptic seizures, the importance of Big Data concepts and techniques, and the practicality of their implementation, has been explored. The use of Big Data enables multimodal research, whose scope and granularity have the potential to change our understanding of prognosis and mortality in epilepsy [78].

To mitigate delays in diagnosis and treatment, researchers have developed an AI-based clinical decision support tool called EpiFinder, which enhances the collection and integration of patient/proxy respondent data [79]. EpiFinder is designed to extract key terms from a patient’s history and incorporate them into a heuristic algorithm that dynamically generates differential diagnoses of epilepsy syndromes. In a recent study, Fergus et al. employed a supervised machine learning algorithm using the KNN classifier in the EEG data to distinguish between seizure and non-seizure epochs without prior knowledge of the focal points of seizures. The model achieved a sensitivity and specificity of 88% and an AUC of 93% for classifying the epochs. Another such study utilized clustering and regression analysis on the spectral and temporal features extracted from the electrographic data, reporting sensitivities of more than 80% [80]. Researchers have also used a novel approach that combines general tensor discriminant analysis (GTDA) followed by KNN on the features extracted from the EEG data, achieving an accuracy of 98% in detecting seizure events [81]. Wang et al. employed principal component analysis (PCA) and analysis of variance on the time and frequency features extracted from EEG data, claiming 99% accuracy for detecting epileptic seizures [82]. Lee et al. used frequency-based feature extraction using PCA, effectively capturing the dynamics of epileptic seizure and reducing false positive rates to only 1.4%, with no false negatives [83].

Visual EEG plots are one of the primary tools used by an epileptologist to detect seizures. To assist in the process, a study developed an artificial visual recognition method of scalp EEG plot images using CNN, which could differentiate seizure versus non-seizure patterns with a median true positive rate of 74% [84]. Another CNN-based study on raw EEG data and frequency domain signals reported an average accuracy of more than 92% for seizure detection across different datasets [85]. Deep neural networks (DNNs) have also been heavily employed by researchers for seizure detection. Hussein et al. used a LSTM network with EEG data, reporting 100% detection accuracy [86]. Another study even enhanced the use of DNN by applying dual DNN on the periodograms of 5 s EEG data, achieving 100% sensitivity and 98% detection accuracy [87]. In another machine learning approach, Guttag et al. employed a SVM model on the features extracted from scalp EEG, and they reported a detection accuracy of 96% [88]. A hybrid model combining a genetic algorithm and particle swarm optimization for optimizing SVM, reported over 99% detection accuracy using EEG data [89]. A comparative study evaluating the performance of several machine learning modalities, including fully connected neural network, Recurrent Neural Network, and CNN, found that CNNs performed the best with an AUC of 0.993 using two-dimensional images of raw EEG as input [90]. It is worth noting that due to inherently noisy nature of EEG signals, using raw data for seizure detection is not always optimal, and researchers have explored various statistical features extracted from EEG and electrooculography (EOG) data to provide valuable information about the underlying neurological mechanisms, in addition to seizure detection [91].

Emerging methods that automatically learn more complex EEG features, such as three-dimensional deep convolution auto-encoder CNN (3D-CNN) architectures, show potential in improving detection in specific datasets [77]. In certain sub-populations, like neonatal EEG, preliminary studies suggests that scaling CNN models may achieve expert level seizure detection performance [92]. Recent advances in Transformers and Attention-based-neural networks [93] are being leveraged for seizure detection to better capture spatio-temporal interrelationships in the signals [94]. While RNNs and LSTMs leverage temporal relationships they still face vanishing gradient problems when processing long sequences. In contrast, transformer-based models offer a much more versatile approach to capture both temporal and spatial interrelationships in long-range contexts, which is the key reason why transformers form the backbone of recent advancement in AI, including Large Language Models, Foundation Models, and Generative AI systems [95]. Table 2 below depicts the time domain, frequency domain, and the wavelet transformation features extracted using EEG and EOG data in different studies for seizure detection. The time-domain features represent statistical measures (e.g., the mean, median, max, min, variance, standard deviation, etc.) derived from a sequence of samples within a specific time window of EEG data measured in milliseconds. Frequency-domain features represent measures (e.g., power, entropy, mean/median frequency, etc.) derived from signal components in the frequency domain, typically using methods like the Fast Fourier Transform. Statistical features such as the mean, median, variance, and standard deviation can be computed in both time and frequency domains. Wavelet transformation features are the parameters (e.g., entropy, relative power, energy, etc.) extracted in the time-frequency domain.

As it can be observed from the table, the performance of the different features used for seizure detection varied between 70% and 100%, depending on the analyzed brain state and the applied AI technique. The most frequently used methods include Support Vector Machines (SVM), Random Forests, Decision Trees, K-Nearest Neighbors (KNN), and deep learning techniques such as Convolutional Neural Networks (CNNs) and Recurrent Neural Networks (RNNs). Overall, these approaches have demonstrated over 90% accuracy in seizure detection, indicating high sensitivity and reliability. In recent years, deep learning algorithms have become increasingly preferred due to their ability to overcome certain limitations of traditional machine learning methods, particularly the issue of model overfitting.

***Seizure classification*.** Transient loss of consciousness (TLC) and seizures have different causes, different diagnoses, and require different treatment. Therefore, the accurate classification of these conditions becomes not only relevant but also clinically significant. In one study, a Random Forest model was applied to the responses of 34 questions on history, patient symptoms, and witness reports. The model was able to classify epilepsy, syncope, and psychogenic nonepileptic (or dissociative seizures) attacks with an accuracy of 86.0% (95% CI = 76.9–92.6%) in 249 patients with TLC [126]. Similarly, Pevy et al. explored the use of audio data recorded from the patients to distinguish epilepsy and psychogenic nonepileptic seizures (PNESs), citing that these two are more difficult to distinguish compared to identifying syncope among the three most common causes of TLC [127]. In another study, the researchers classified seven variants of seizures with non-seizure EEG through the application of CNNs and transfer learning [128]. The model achieved a multi-class classification accuracy of 88.3% across seizure types, including simple partial, complex partial, focal non-specific, generalized non-specific, absence, tonic, tonic–clonic, and non-seizure events. Another study used graph CNNs with a recurrent network and self-supervised pre-training to detect and classify seizure types using a publicly available large dataset. This method demonstrated an improved accuracy over conventional CNNs, and importantly improved the classification of rare seizure types [129]. Saputro et al. employed Support Vector Machine to distinguish between focal non-specific, generalized non-specific, and tonic–clonic seizures. Using the features, Mel Frequency Cepstral Coefficients (MFCC), and Hjorth descriptor, they were able to achieve an average classification sensitivity of 90.25%, average specificity of 97.83% and average accuracy of 94% [130]. The spike wave discharges have been used to predict the impaired consciousness in absence epilepsy. Using both time- and frequency-domain features, Springer et al. employed linear discriminator analysis (LDA) and SVM to classify between spared and impaired behavior in epileptic patients. They reported a 100% rejection of minimal false discovery rate for both classifiers. Moreover, for labeled data, the LDA achieved a sensitivity of 93% while the SVM reached 91% [131]. A study using a wearable accelerometer-based system evaluated the classification accuracy of tonic–clonic seizures in epilepsy patients, using KNN, Random Forest, and SVM algorithms. The highest sensitivity was reported to be that of the KNN (100%, 0.05 false positives/hour), while the lowest sensitivity was observed for Random Forest (90%, 0.01 FP/h) [132]. Similarly to the studies for seizure predictions, for the applications discussed above, deep learning methods exceed the traditional machine learning approaches due to several advantages including larger amounts of training data, the automated extraction of relevant features, hierarchical data representation, and adaptability (flexibility) [133].

## 4. Application of Assistive AI in Clinical Care for LMICs

One of the primary challenges to the proper diagnosis of epilepsy is the lack of experts qualified to read EEG data. This shortage is not only present in LMICs, resulting in 90% of individuals with epilepsy receiving no treatment, but also in HICs like the United Kingdom, where charities such as tele-EEG support tele-readers to circumvent the problem [51]. Moreover, it has been shown that the most common sources of error in EEG readings occur due to the lack of adequate training among neurologists, who may lack the specialized expertise to read EEG at the same level as an epilepsy specialist [134]. These challenges may be addressed through the integration of artificial intelligence (AI), which can assist clinicians by improving the sensitivity and specificity of EEG analyses and support diagnostic accuracy [135]. Researchers have tested the use of low-cost EEG systems in rural settings to evaluate their efficacy. For instance, Sokolov et al. deployed a tablet-based EEG system with a 14-electrode cap in Guinea and showed that it maintained reproducible signal quality across repeated testing and was effective for the detection of epileptiform discharges [136]. Similarly, other studies have compared smartphone-based EEG [137], built on the Smartphone Brain Scanner2 (SBS2) which uses a 14-electrode EasyCap headset (approx. cost of 300 USD) connected wirelessly to an android tablet [138], to a standard clinical EEG in detecting epileptiform abnormalities. They found that the SBS2 had low to moderate sensitivity but high specificity in detecting epileptiform abnormalities compared to clinical EEG. In such settings, the use of AI could significantly enhance the processing speed and seizure detection, classification, or prediction capabilities, as discussed in the previous sections.

Anticipating and predicting seizures is crucial, as it has been evidenced that the seemingly random nature of the seizure occurrence considerably increases the morbidity and mortality risks of epilepsy [139]. Various studies have explored approaches to predict seizure occurrence. One such study showed that patients experience prodromal symptoms, with early warning signs and awareness when seizures are more likely to occur, and it reported increased cerebral blood flow around 10 min prior to temporal lobe seizure [140]. Such findings present a perfect avenue for testing some AI-based algorithms in developing non-EEG-based wearable devices to predict such physiological markers (e.g., variations in blood flow). There has been limited research in detecting epilepsy using EEG during seizure-free periods (or interictal periods). Recent studies using machine learning and deep learning algorithms have shown encouraging results in this area. For example, a recent study used specific epileptic EEG sub-bands to predict epilepsy occurrence using seizure-free data with a 99% accuracy [141]. However, one should note that the high accuracy in the study was likely because of the intracranial single-channel short EEG segments. Another study adopted a more practical approach of applying non-invasive 21-channel EEG recordings and obtained a decent 75% accuracy in detecting epileptic episodes [142]. Similarly, by using brain network structural and functional connectivity measures and combining them with machine learning models, other studies have been able to predict epileptic onset from seizure-free periods or improve the sensitivity of EEG-based expert visual diagnosis [143,144]. For instance, Cao et al. used interictal seizure-free video-EEGs to first estimate functional connectivity using mutual information and coherence correlation and utilized them as features in a KNN model. The model achieved a 97% classification accuracy for epilepsy patients when compared to healthy controls and an accuracy of 73% for when compared to patients with non-epileptic events [145]. In a similar study, researchers extracted 4 s segments of EEG recordings from epilepsy patients and extracted 532 segments of epileptiform discharges to compare them with 100 segments from healthy controls. Using a Random Forest classifier, they were able to determine epileptic EEG signals with an accuracy of over 98% [146]. These examples noticeably emphasize the usefulness of an AI-based data analytic framework that could be suitable for clinical settings in a resource-limited environment.

However, it is important to acknowledge the systemic limitations of LMIC health systems that may hinder the effective implementation of these promising assistive AI tools. Key limitations are poor and intermittent power supply [147], unreliable internet connectivity [148], and poor health system resilience [149]. Additionally, the potential incompatibility of these assistive AI tools with existing paper-based and/or electronic health systems and the limited digital literacy among healthcare workers pose significant barriers [150] in integrating these tools within national health management information systems. Apart from these limitations, assistive AI tools may bring additional challenges. For example, adopting these tools may further strain already overburdened health workers [151]. Similarly, weak data infrastructure in many LMICs increases the risk of data privacy breaches [152]. In this context, a phased approach, starting with pilot programs in feasible regions and gradual adaptation and expansion based on lessons learned, could help ensure the effective and sustainable use of assistive AI tools.

## 5. The Socio-Economic Impact of Assistive AI for Epilepsy in LMICs

Assistive AI has the potential to bring impactful change in the diagnosis and treatment of epilepsy, ultimately improving both health and socio-economic outcomes in LMICs. One of its most significant contributions lies in enhancing diagnostic accuracy. This could help reduce the diagnostic gap in LMICs and minimize the risk of misdiagnosis. As discussed earlier, there is a severe lack of neurologists and epileptologists in LMICs. With the help of algorithms developed through assistive AI, primary care physicians, who are widely available, can diagnose epilepsy more quickly and with greater accuracy. Shifting diagnostic tasks from scarce epileptologists to relatively abundant primary care physicians can significantly help reduce the diagnostic gap. Moreover, given the higher disparity in the distribution of diagnostic facilities between rural and urban areas within a country, assistive AI can also help reduce health inequities in LMICs. Furthermore, the burden of misdiagnosis can be astonishingly high, especially for the patient, and it can manifest in the form of stigma, unnecessary exposure to AEDs, and drug resistance [153]. Improved diagnostic precision through AI can help prevent such detrimental consequences related to misdiagnosis. Second, beyond supporting clinical decision making, assistive AI can also increase confidence among healthcare workers. For example, a scoping review on clinical decisions using m-Health in sub-Saharan Africa found that a mobile clinical decision support system increased self confidence among healthcare workers and reduced their reliance on peers or referral facilities. From a doctor–patient relationship perspective, some studies have reported that it can help build trust between patients and healthcare providers [154]. Third, assistive AI can substantially reduce the time and cost associated with diagnosing epileptic seizures. Usually, neurologists or epileptologists analyze the recorded EEG through visual inspection to trace the patterns of epilepsy across time series [155]. Since EEG data are non-linear and non-stationary, this process is usually lengthy and inefficient, especially when the experts must analyze long-term EEG recordings to accurately diagnose patterns of seizure. For example, recent evidence suggests that the use of deep learning could reduce the need for human review by 65 – 99% depending on the required level of diagnostic precision [155]. More importantly, with the growing use of low-cost portable EEG systems, the diagnosis of epilepsy could become much cheaper, increasing affordability among the poor population in LMICs. With evidence for cost savings and improvements in efficiency associated with task-shifting strategies [156], the diagnosis of epilepsy by trained primary healthcare workers instead of epileptologists or neurologists could directly reduce out-of-pocket expenditure for patients and save costs for the overall health system in LMICs.

## 6. Future Directions and Conclusions

In this review, we examined the application of artificial intelligence (AI) in clinical settings for the diagnosis and management of epilepsy. Given the numerous challenges associated with providing medical care for epilepsy patients in low- and middle-income countries (LMICs), we posit that the development and implementation of sensitive, accessible, adaptable, and low-cost AI-based tools could significantly improve diagnostic workflows and help alleviate the burden of epilepsy in these regions. The growing body of evidence highlights a transformation in how epilepsy is managed across clinical contexts, whether in high-income countries (HICs) or LMICs. However, only a few of these innovations have successful transitioned into the actual clinical settings of LMICs. Barriers include the cost of EEG devices, long-term maintenance challenges, a shortage of trained personnel, and limited awareness that perpetuates epilepsy-related stigma. On the one hand, there is an urgent need to generate more robust evidence on the effectiveness of AI-assisted diagnostic tools in epilepsy. On the other, it is essential to carefully weigh the unintended consequences associated with deploying such technologies [157]. While AI has shown considerable promise in controlled studies, the large-scale validation of the generalization, utility in LMICs settings, and trustworthiness of these systems is still understudied. Generalizing the higher accuracy results obtained in curated dataset to the real-world data in prospective, diverse clinical settings is a crucial research priority [62,158]. While some reviews have focused on what the pathway to the clinical translation of these AI tools could be, they are still focused on HICs settings [62] and do not directly translate to LMIC health settings.

Telemedicine-specific solutions are also highly context-dependent and must be tailored to the unique infrastructural and policy environments of LMICs. Hence, dedicated research to identify context-specific challenges and needs is necessary to inform the integration of AI and telemedicine into epilepsy care in LMICs [159]. Although regional and national plans in improving epilepsy care have emphasized the importance of the capacity building of primary care centers and integrating innovative technologies [160], AI- and EEG-specific details on how they could be integrated into such plans needs further investigation. In this context, trustworthy AI and regulatory frameworks should be explored in using AI for epilepsy in various settings. This should be built based on the existing body of work such as FUTURE AI, which offers principled frameworks and practical guidelines on the design, development, and deployment of AI in healthcare [161]. However, such general frameworks need specialization in the following two specific directions: (1) epilepsy-specific considerations, recognizing the unique challenges faced by people living with epilepsy; and (2) health system contextualization, accounting for regulatory environments and socio-cultural factors such as stigma, access to care, and support for people with disabilities [162].

A multifaceted approach is essential to address these interrelated aspects. One avenue is the development and testing of low-cost hardware solutions. For example, *BioAmp EXG Pill* developed by a startup (*Upside Down Labs*) in India with a starting price of $60 that can record high-quality biosignals from the heart (electrocardiography), brain (EEG), eyes (EOG), and muscles (electromyography), and it may offer affordable, scalable options for LMICs. Another key direction involves leveraging edge AI and low-resource models, where algorithms can run locally on inexpensive hardware (e.g., low-cost GPUs or embedded processors). Lightweight deep learning techniques, particularly those optimized for mobile or resource-constrained environments, show promise in this direction for supporting epilepsy diagnosis and management in LMICs. One example is the application of automated EEG analysis using compact Convolutional Neural Networks (CNNs) embedded into portable, affordable EEG systems to expand access to timely care while reducing dependence on specialized infrastructure and personnel. This is particularly relevant in LMICs, where the infrastructure for maintaining large-scale centralized servers is often lacking. Additionally, there is a need for an open access, collaborative platform to unify ongoing work on AI-based tools. Such a platform would allow researchers to contribute, share, and refine algorithms in real time, while also offering easy access for clinicians, healthcare workers, and epileptologists. This collective effort could accelerate innovation and translation into practice. And finally, alongside technological development, efforts should be placed in educating communities about the disease and the stigmas around it. Presenting educational materials and research findings through targeted outreach in the form of blogs, newspaper articles, social media video clips, podcasts, public outreach, etc., would be the first steps for researchers. Funding agencies should also prioritize educational and community engagement proposals, recognizing their key role in the successful implementation and sustainability of AI tools in epilepsy care.

## Figures and Tables

**Table 1:** Comparative analysis of epilepsy metrics between high-income and low- and middle-income countries.

Metric	High-Income Countries	Low- and Middle-Income Countries
Annual new epilepsy cases per 100,000 population	49	139
Lifetime prevalence of epilepsy per 1000 population	5.18	8.75
Median point prevalence of epilepsy per 1000 population	5.49	6.68
Annual epilepsy-related deaths	Less than 20% of 125,000	More than 80% of 125,000

**Table 2:** Features extracted from EEG and EOG data and used for seizure detection.

Features	Brain State	Technique	Performance	Citations
**Time-domain features**				
Mean	Pre-ictal/ictal	Decision forest	Average accuracy: 98.5–99.7%	[96]
	Pre-ictal/ictal	Random forest	Sensitivity: 93.8%	[97]
	Pre-ictal/ictal/interictal	Random forest	Accuracy: 94.3%	[98]
	Ictal	Random forest	Area under the ROC curve: 0.99	[99]
	Ictal/interictal	Random forest	Area under the ROC curve: 0.90	[100]
	Ictal/interictal	Random forest	Average accuracy: 98.6%	[101]
	Ictal	Decision forest	Area under the ROC curve: 0.64	[102]
	Ictal	Support vector machine	Accuracy: 99.4%	[103]
		K-nearest neighbors	Accuracy: 99.4%	
Root mean square	Ictal/interictal	Support vector machine	Accuracy: 95.6%	[104]
	Ictal/interictal	Support vector machine	Accuracy: 99.1%	[105]
	Ictal/interictal	K-nearest neighbors	Area under the ROC curve: 0.91	[106]
Variance	Ictal/interictal	Support vector machine	Accuracy: 95.6%	[104]
	Pre-ictal, ictal	Random forest	Sensitivity: 93.8%	[97]
	Ictal/interictal	Support vector machine	Accuracy: 99.1%	[105]
	Ictal/interictal	K-nearest neighbors	Area under the ROC curve: 0.91	[106]
	Ictal/interictal	Random forest	Area under the ROC curve: 0.90	[100]
Maxima and minima	Ictal/interictal	Support vector machine	Accuracy: 99.1%	[105]
	Ictal/interictal	Random forest	Average accuracy: 98.6%	[101]
	Ictal	Support vector machine	Accuracy: 99.4%	[103]
		K-nearest neighbors	Accuracy: 99.4%	
	Pre-ictal/ictal	Decision forest	Average accuracy: 98.5–99.7%	[96]
	Ictal	Decision forest	Area under the ROC curve: 0.67	[102]
Mode and median	Ictal/interictal	Random forest	Average accuracy: 98.6%	[101]
Skewness	Pre-ictal/ictal	Decision forest	Average accuracy: 98.5–99.7%	[96]
	Ictal	Support vector machine	Accuracy: 99.4%	[103]
		K-nearest neighbors	Accuracy: 99.4%	
	Ictal/interictal	Random forest	Average accuracy: 98.60%	[101]
	Pre-ictal/ictal/interictal	Random forest	Accuracy: 94.3%	[98]
	Pre-ictal/ictal	Random forest	Sensitivity: 93.8%	[97]
	Ictal/interictal	Support vector machine	Accuracy: 99.1%	[105]
	Ictal/interictal	K-nearest neighbors	Area under the ROC curve: 0.91	[106]
	Ictal/interictal	Random forest	Area under the ROC curve: 0.90	[100]
Kurtosis	Pre-ictal/ictal	Decision forest	Average accuracy: 98.5–99.7%	[96]
	Ictal	Support vector machine	Accuracy: 99.4%	[103]
		K-nearest neighbors	Accuracy: 99.4%	
	Ictal/interictal	Random forest	Average accuracy: 98.6%	[101]
	Pre-ictal/ictal/interictal	Random forest	Accuracy: 94.3%	[98]
	Ictal/interictal	Support vector machine	Accuracy: 99.1%	[105]
	Ictal/interictal	K-nearest neighbors	Area under the ROC curve: 0.91	[106]
	Ictal/interictal	Random forest	Area under the ROC curve: 0.90	[100]
Line length	Pre-ictal/ictal	Decision forest Support vector machine K-nearest neighbors Random forest Random forest Random forest Support vector machine Decision forest Neural network Burst detection algorithm Multi-layer perceptron neural network	Average accuracy: 98.5–99.7% Accuracy: 99.4% Accuracy: 99.4% Area under the ROC curve: 0.90 Accuracy: 94.3% Sensitivity: 93.8% Area under the ROC curve: 0.88 Area under the ROC curve: 0.77 -Accuracy: 84.2% Accuracy: 99.6%	[96] [103] [100] [98] [97] [107] [102] [108] [109] [110]
	Ictal			

	Ictal/interictal			
	Pre-ictal/ictal/interictal			
	Pre-ictal/ictal			
	Ictal/interictal			
	Ictal			
	Ictal			
	Ictal			
	Ictal			
	Ictal			
Energy	Ictal	Decision forest	Area under the ROC curve: 0.74	[102]
	Ictal/interictal	Support vector machine	Accuracy: 99.4%	[103]
		K-nearest neighbors	Accuracy: 99.4%	
	Ictal	Independent component analysis	Area under the ROC curve: 0.92	[111]
	Ictal	Support vector machine	Accuracy: 95.6%	[104]
	Ictal	Automated classification algorithm	Accuracy: 99.4%	[112]
	Ictal/interictal	Support vector machine	Accuracy: 99.1%	[105]
	Pre-ictal/ictal	Decision forest	Average accuracy: 98.5–99.7%	[96]
Power	Ictal	Decision forest	Area under the ROC curve: 0.74 Area under the ROC curve: 0.99	[102] [99]
	Ictal	Random forest		
Shannon entropy	Ictal/interictal	Support vector machine	Accuracy: 99.5%	[113]
	Ictal/interictal	Random forest	Average accuracy: 98.6%	[101]
	Ictal	Support vector machine	Accuracy: 99.4%	[103]
		K-nearest neighbors	Accuracy: 99.4%	
	Pre-ictal, ictal	Decision forest	Average accuracy: 98.5–99.7%	[96]
Sample and approximate entropies	Ictal	K-nearest neighbor	Accuracy: 98.0%	[114]
	Ictal	Discrete wavelet transformation	Accuracy: 98.0%	[115]
	Ictal/interictal	Extreme learning machine	Accuracy: 95.6%	[116]
	Ictal/interictal	Extreme learning machine	Accuracy: 99.6%	[117]
		Support vector machine	Accuracy: 100%	
	Pre-ictal	Fuzzy Sugeno Classifier	Accuracy: 98.1%	[118]
	Ictal/interictal	Support vector machine	Accuracy: 99.1%	[105]
	Ictal/interictal	K-nearest neighbors	Area under the ROC curve: 0.91	[106]
Fuzzy entropy	Ictal/interictal	Support vector machine	Accuracy: 99.5%	[113]
Hurst exponent	Ictal/interictal	Random forest	Average accuracy: 98.6%	[101]
Standard deviation	Pre-ictal, ictal	Decision forest	Average accuracy: 98.5–99.7%	[96]
	Ictal	Random forest	Area under the ROC curve: 0.99	[99]
	Pre-ictal/ictal/interictal	Random forest	Accuracy: 94.3%	[98]
	Ictal/interictal	Support vector machine	Accuracy: 99.1%	[105]
	Ictal/interictal	Random forest	Average accuracy: 98.6%	[101]
	Ictal	Support vector machine	Accuracy: 99.4%	[103]
		K-nearest neighbors	Accuracy: 99.4%	
Autocorrelation	Pre-ictal, ictal	Random forest	Sensitivity: 93.8%	[97]
	Ictal/interictal	Random forest	Area under the ROC curve: 0.90	[100]
Mean absolute deviation	Ictal/interictal	Random forest	Area under the ROC curve: 0.90	[100]
Amplitude	Ictal/interictal	Extreme learning machine	Sensitivity: 97.7%	[119]
Pattern match regularity statistic	Ictal/interictal	Extreme learning machine	Sensitivity: 97.7%	[119]
**Frequency-domain features**				
Spectral power	Pre-ictal/ictal	Random forest	Sensitivity: 93.8%	[97]
	Ictal	Random forest	Sensitivity: 80.8%	[120]
	Ictal	Artificial neural network	F-measure: 0.82	[121]
	Ictal/interictal	Support vector machine	Accuracy: 99.1%	[105]
	Ictal/interictal	Artificial neural network	Accuracy: 97.7–100%	[122]
Spectral entropy	Ictal/interictal	Support vector machine	Accuracy: 99.1%	[105]
Peak frequency	Ictal/interictal	K-nearest neighbors	Area under the ROC curve: 0.91	[106]
Median frequency	Ictal/interictal	Support vector machine	Accuracy: 99.1%	[105]
	Ictal/interictal	K-nearest neighbors	Area under the ROC curve: 0.91	[106]
Power spectral density	Ictal	Random forest	Sensitivity: 80.8%	[120]
	Ictal/interictal	Extreme learning machine	Sensitivity: 97.7%	[119]
Average power and power ratio	Ictal/interictal	Random forest	Area under the ROC curve: 0.90	[100]
Mean frequency	Ictal/interictal	Support vector machine	Accuracy: 96.1%	[123]
Total spectral power	Ictal	Random forest	Sensitivity: 80.8%	[120]
	Ictal	Artificial neural network	F-measure: 0.82	[121]
Root mean square bandwidth	Ictal/interictal	Support vector machine	Accuracy: 96.1%	[123]
Discrete cosine transform	Ictal/interictal	Support vector machine	Accuracy: 84.1%	[124]
**Wavelet transformation features**				
**DWT features**				
Bounded variation	Ictal	Decision forest	Area under the ROC curve: 0.53	[102]
Coefficients	Ictal	Decision forest	Area under the ROC curve: 0.66	[102]
	Interictal	K-nearest neighbors	Accuracy: 98.0%	[125]
	Ictal/interictal	Support vector machine	Accuracy: 84.1%	[124]
Energy	Ictal	Decision forest	Area under the ROC curve: 0.71	[102]
	Interictal	K-nearest neighbors	Accuracy: 98.0%	[125]
Relative power	Interictal	K-nearest neighbors	Accuracy: 98.0%	[125]
	Ictal	Decision forest	Area under the ROC curve: 0.81	[102]
Entropy	Ictal	Decision forest	Area under the ROC curve: 0.71	[102]
Relative bounded variation	Ictal	Decision forest	Area under the ROC curve: 0.54	[102]
Relative scale energy	Ictal	Decision forest	Area under the ROC curve: 0.61	[102]
**CWT features**				
Energy standard deviation	Ictal	Decision forest	Area under the ROC curve: 0.70	[102]
Coefficient z-score	Ictal	Decision forest	Area under the ROC curve: 0.69	[102]

CWT: continuous wavelet transformation; DWT: discrete wavelet transformation; and ROC: receiver operating characteristic.
